# Association of Loneliness and Wisdom With Gut Microbial Diversity and Composition: An Exploratory Study

**DOI:** 10.3389/fpsyt.2021.648475

**Published:** 2021-03-25

**Authors:** Tanya T. Nguyen, Xinlian Zhang, Tsung-Chin Wu, Jinyuan Liu, Collin Le, Xin M. Tu, Rob Knight, Dilip V. Jeste

**Affiliations:** ^1^Department of Psychiatry, University of California, San Diego, La Jolla, CA, United States; ^2^Sam and Rose Stein Institute for Research on Aging, University of California, San Diego, La Jolla, CA, United States; ^3^Veterans Affairs San Diego Healthcare System, La Jolla, CA, United States; ^4^Center for Microbiome Innovation, University of California, San Diego, La Jolla, CA, United States; ^5^Department of Family Medicine and Public Health, University of California, San Diego, La Jolla, CA, United States; ^6^Department of Pediatrics, University of California, San Diego, La Jolla, CA, United States; ^7^Department of Computer Science and Engineering, University of California, San Diego, La Jolla, CA, United States; ^8^Department of Bioengineering, University of California, San Diego, La Jolla, CA, United States; ^9^Department of Neurosciences, University of California, San Diego, La Jolla, CA, United States

**Keywords:** microbiome, social isolation, compassion, social behavior, gut-brain-axis, bacteria

## Abstract

Loneliness and wisdom have opposite effects on health and well-being. Loneliness is a serious public health problem associated with increased morbidity and mortality. Wisdom is associated with better health and well-being. We have consistently found a strong negative correlation between loneliness and wisdom. The present study aimed to investigate the association of loneliness and wisdom with the gut microbiome. One hundred eighty-four community-dwelling adults (28–97 years) completed validated self-report-based measures of loneliness, wisdom, compassion, social support, and social engagement. Fecal samples were collected and profiled using 16S rRNA sequencing. Linear regression analyses, controlling for age and body mass index, revealed that lower levels of loneliness and higher levels of wisdom, compassion, social support, and social engagement were associated with greater phylogenetic richness and diversity of the gut microbiome. Partial least squares (PLS) analysis to investigate multivariate relationships extracted two composite variables. Linear regression model predicting alpha-diversity with PLS components revealed that a linear combination of all psychosocial predictors (with negative loading for loneliness and positive loadings for all others, including wisdom, compassion, social support, and social engagement) was significantly associated with alpha-diversity. For beta-diversity, compassion and wisdom accounted for a significant proportion of variance in overall microbial community composition. Findings may have implications for interventions to reduce loneliness and possibly its health-related adverse consequences. Future research should explore whether increasing compassion and wisdom may improve loneliness and overall well-being as well as microbial diversity.

## Introduction

Loneliness and social isolation are important public health risks, linked to worse emotional, cognitive, and physical health, functional decline, and premature death (1, 2). Loneliness is the subjective negative experience that results from a discrepancy between one's preferred and actual social relationships, whereas social isolation is the objective state of having few or infrequent social connections. Loneliness is generally conceptualized as having an acute, transient form or a persistent, stable form (3). The latter—i.e., persistent or chronic loneliness, rather than short-term fluctuations—is considered to be biologically toxic and is the focus in the present paper. Both loneliness and social disconnectedness are independently associated with worse physical health; however, the mechanisms responsible are not fully understood. Loneliness is associated with changes in cardiovascular, neuroendocrine, and immune function, including elevations in pro-inflammatory biomarkers and activation of the hypothalamic-pituitary-adrenal axis (1)—biological pathways that are associated with the microbiota-gut-brain-axis. On the other hand, wisdom, social support, and social engagement are associated with greater well-being and health (4, 5). Loneliness has been consistently found to be strongly inversely correlated with wisdom (6–8).

Wisdom is a multifaceted human characteristic with affective (or compassionate), reflective, and cognitive dimensions (9, 10). The affective dimension refers to the presence of positive emotions and behaviors toward others, such as empathy and acts of compassion. The reflective dimension is the ability to engage in reflective thinking and development of self-awareness. The cognitive dimension refers to one's knowledge about the world and comprehension of the deeper meaning of life events. Of the dimensions or components of wisdom, pro-social behaviors or compassion is most predictive of loneliness (6). Though wisdom has traditionally been viewed as a construct restricted primarily to philosophy or religion, empirical research in recent years has demonstrated that wisdom is partially influenced by biology (11, 12). Studies in behavioral genetics and neurobiology (13–16) suggest strong genetic and biological components of both loneliness and wisdom that underscore their evolutionary value (11, 17, 18) and public health implications, including their potential relationship with the gut microbiome.

The ability of gut microbes to communicate with the brain and modulate human behavior has emerged as an exciting concept in health and disease. The “microbiota-gut-brain-axis” involves bi-directional signaling between the gastrointestinal and central nervous systems and is regulated at neural, hormonal, and immunological levels (19). Alterations of these systems can result in disruptions of stress-response and behavior, from emotional arousal, affective behavior, and motivation, to higher-order cognitive functions such as decision-making (20). Studies in humans have found that the gut microbiota is associated with personality traits, such as neuroticism, openness, agreeableness, and conscientiousness, and psychological constructs, including subjective stress, self-compassion, affective empathy, and emotional well-being (21–24). Notably, compassion and empathy are important components of wisdom, and the putative neurocircuitry of wisdom overlap with structures that have been implicated in the microbiota-gut-brain axis, including fronto-limbic networks (25). Additionally, recent research has revealed connections between the gut microbiome and social behavior, and it has been proposed that the microbiome may be important to host sociality, particularly in the context of evolutionary-based theories of the benefits of mutualism in social survival (26–29). Social behavior and interactions can affect the composition of the gut microbiota (30, 31). Conversely, animal experiments have shown that gut microbes produce chemical signal used in social communication (32, 33) and that presence of the gut microbiota is necessary for social motivation (34). In humans, people with larger social networks tend to have more diverse gut microbiotas (22); however, the nature of and pathways that mediate this association are yet to be fully elucidated. Low microbial alpha-diversity has been associated with a number of diseases including obesity, inflammatory bowel diseases, and neurological and psychiatric disorders (35–37). No study to our knowledge has investigated gut microbial diversity with loneliness and wisdom. An investigation into the biological mechanisms underlying these psychological constructs is important to potential understanding of how loneliness may contribute to physical morbidity/mortality.

The present study sought to examine the relationship of microbial alpha-diversity and beta-diversity to loneliness and wisdom, as well as related psychosocial factors, in a sample of community-dwelling individuals across the adult lifespan. We hypothesized that higher levels of loneliness and lower levels of wisdom, compassion, social support, and social engagement would be associated with lower microbial diversity.

## Method

### Participants

The study included 184 community-dwelling adults (28–97 years). Inclusion criteria were age between 21 and 100 years, English fluency, and physical/cognitive ability to complete study assessments. This investigation was part of larger ongoing studies, including the SAGE (Successful AGing Evaluation) study and non-psychiatric comparison subjects from a study of aging and mental illness (38–40). Participants in the SAGE study were recruited using list-assisted random digit dialing of adults living in San Diego County, CA. The SAGE study originally targeted adults aged 50–100 years but was subsequently expanded to include participants aged 21–100 years to add younger individuals (39). Participants completed 25-min structured telephone interview that included questions about general health, depression and anxiety, and cognitive functioning. Exclusion criteria were residence in a nursing home or requiring daily skilled nursing care, diagnosis of dementia, or terminal illness or requiring hospice care. Non-psychiatric comparison participants from the study of aging and mental illness (38, 40) were recruited through multiple methods, including recruitment flyers in the community, www.ResearchMatch.org, and word-of-mouth. Participants were screened for major neuropsychiatric disorders using the Mini-International Neuropsychiatric Interview (MINI) (41). Exclusion criteria were past or present diagnosis of a major DSM-IV-TR Axis I diagnoses, alcohol or other substance abuse or dependence within 3 months prior to enrollment, or diagnosis of dementia, intellectual disability disorder, or a major neurological disorder. Research protocols were approved by the UCSD Human Research Protections Program. All participants provided informed consent to participate.

### Clinical Assessments

Participants completed validated scales of loneliness (UCLA Loneliness Scale) (42), wisdom (including cognitive, affective, and reflective dimensions; Three-Dimensional Wisdom Scale) (9), compassion (Santa Clara Brief Compassion Scale) (43), social support (Emotional Support Scale) (44), and social engagement (Cognitively Stimulating Questionnaire) (45). Additionally, depression (Center for Epidemiologic Studies Depression Scale) (46) and physical well-being (Medical Outcomes Study Health Survey) (47) were assessed.

### Fecal Sample Collection and Analysis

Fecal samples were obtained from participants using at-home self-collection kits (BD SWUBE Dual Swab Collection System; BD Worldwide) and returned via mail. Samples were immediately frozen and stored at −80°C. DNA extraction and 16S rRNA amplicon sequencing were completed using the Earth Microbiome Project protocols (48, 49). In brief, DNA was extracted using the Qiagen MagAttract PowerSoil DNA kit (50). The 16S rRNA gene was amplified using unique reverse barcoded primers targeting the V4 region, and sequenced on Illumina MiSeq or HiSeq 4000 platforms, yielding paired-end 150 base-pair reads (median 17,160 reads/sample) (51, 52).

Sequencing data were processed using QIIME2 (version 2019.7) (53, 54). Raw sequences were demultiplexed and processed using Deblur (55), and previously recognized bloom sequences were removed (56). Deblur amplicon sequence variants were inserted into Greengenes 16S rRNA gene tree using SEPP (57, 58). Taxonomy was assigned using a pre-trained Naïve Bayes classifier (59). The output feature table was rarefied to 5,000 sequences per sample. Microbial community structure was characterized using measures of alpha-diversity and beta-diversity. Alpha-diversity is the ecological diversity (i.e., richness, evenness, compositional complexity) of a single sample and was quantified using Faith's Phylogenetic Diversity (PD), which measures the total length of branches in a reference phylogenetic tree for all species in a given sample (60). Beta-diversity measures the similarity (or dissimilarity) of microbial community composition between samples. Beta-diversity was characterized using Aitchison distance, a metric rooted in a centered log-ratio transformation and matrix completion called robust principal components analysis (PCA) that accounts for the sparse compositional nature of microbiome data sets (61).

### Statistical Analysis

A two-sided alpha = 0.05 was used to determine statistical significance. The adaptive control of false discovery rate (FDR) procedure by Benjamini-Hochberg was used for multiple comparisons (62). Univariate linear regression models were first used to examine the relationship between alpha-diversity and psychosocial variables, controlling for age and body mass index (BMI). Associations between psychosocial variables and beta-diversity were performed using ADONIS (63) with significance calculated using PERMANOVA with 999 permutations (64). Subsequently, we used partial least squares (PLS) regression to investigate the multivariate relationship of all psychosocial variables and alpha-diversity in a single model. PLS constructs a series of composite variables that are linear combinations of the predictors such that the composite variables extract the most information from the predictors (i.e., has high variance, as is the case in PCA) and, at the same time, have high correlation with the response (which is not achieved in PCA) (65). We did not run PLS on beta-diversity since multivariable regression models require independence among dependent variables given the predictors, which is not the case for beta-diversity. For exploratory analyses, we examined age and gender as potential moderating factors by modeling an interaction term between psychosocial predictors on alpha- and beta-diversity in analyses.

## Results

Demographic and clinical characteristics for the sample are presented in Table 1.

**Table 1:** Demographic and clinical characteristics of the sample.

	**Total sample (*N* = 184)**
Age (years)	62.39 (15.77)
Gender (female)	89 (48%)
Race/Ethnicity
Caucasian	140 (76%)
Hispanic	25 (14%)
Asian	9 (5%)
African American	8 (4%)
Native American and Other	2 (1%)
BMI	26.43 (5.21)
Loneliness (UCLA-3)	35.86 (10.39)
Wisdom (3D-WS)	
Cognitive dimension	3.52 (0.53)
Reflective dimension	3.96 (0.51)
Affective dimension	3.40 (0.52)
Total score	3.62 (0.43)
Compassion (SCBCS)	4.73 (1.32)
Social support (ESS)	2.65 (0.55)
Social engagement (CSQ)	2.17 (0.61)

*Data are presented as mean (standard deviation) for continuous variables or n (percent) for categorical variables*.

*3D-WS, Three-Dimensional Wisdom Scale; BMI, body mass index; CSQ, Cognitively Stimulating Questionnaire; df, degrees of freedom; ESS, Emotional Support Scale; SCBCS, Santa Clara Brief Compassion Scale; UCLA-3, UCLA Loneliness Scale, Version 3*.

### Alpha-Diversity

#### Univariate Analysis

**Figure 1:**
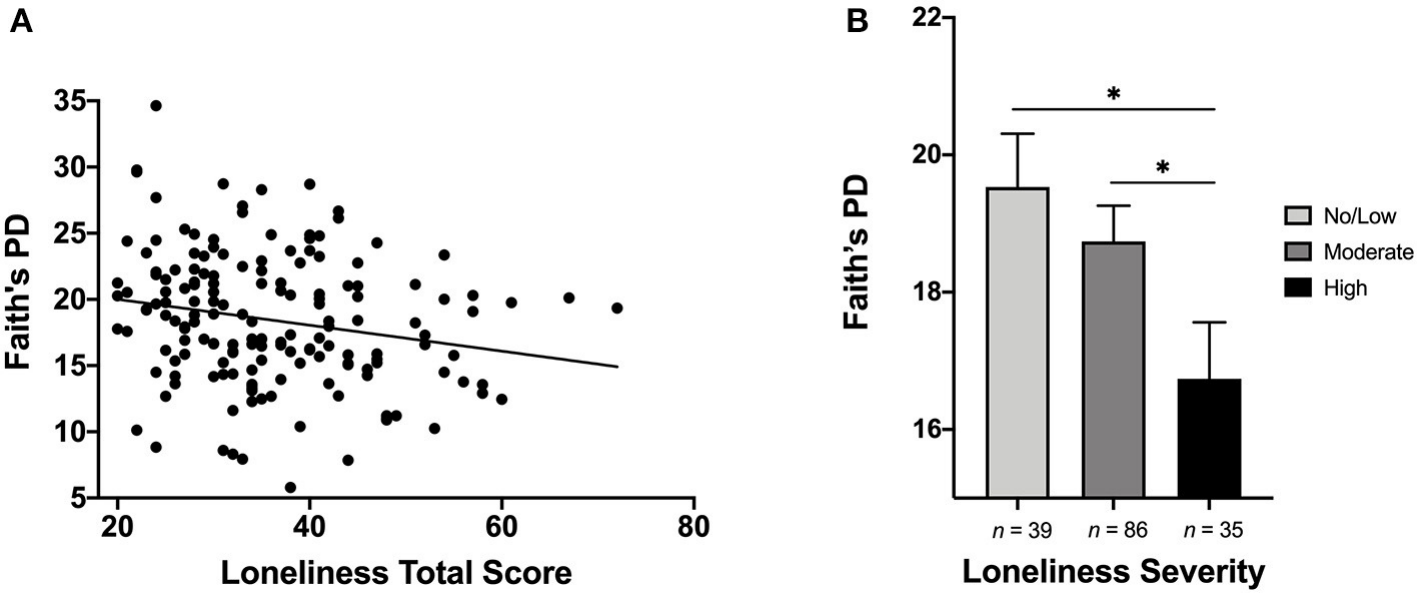
**(A)** Scatterplot depicting the relationship between loneliness and alpha-diversity (Faith's Phylogenetic Diversity). Higher level of loneliness was associated with significantly lower alpha-diversity. **(B)** Bar plot illustrating differences in alpha-diversity based on loneliness severity categories. Individuals with High levels of loneliness exhibited significantly lower gut microbial alpha-diversity, compared to those with No/Low and Moderate levels of loneliness. *p or *q* < 0.05.

Results of linear regression models indicated significant associations between alpha-diversity and loneliness, wisdom, social support, compassion, and participation in social engagement activities (Table 2). Lower levels of loneliness and higher levels of wisdom (total score and reflective and affective dimensions), compassion, social support, and social engagement were associated with greater alpha-diversity of the gut microbiome (Figure 1A). Effect sizes were small to medium. We also examined group differences in alpha-diversity based on loneliness severity categories, given that there are established cut-offs for loneliness severity: total score <28 = No/Low, 28–43 = Moderate, and >43 = High (66). Alpha-diversity significantly differed across severity categories (*F* = 3.27, *p* = 0.041), with lower alpha-diversity among individuals with high loneliness severity, compared to those with no/low (*p* = 0.015) and moderate (*p* = 0.042) severity (Figure 1B).

**Table 2:** Coefficients from univariate linear regression models of each psychosocial factor predicting alpha-diversity (Faith's Phylogenetic Diversity), after controlling for age and body mass index.

	**β**	***t***	***p***	***q*^†^**	**Partial ***η^2^*****
Loneliness (UCLA-3)	−0.170	−2.095	0.038^*^	0.021^*^	0.028
Wisdom (3D-WS)					
Cognitive dimension	0.012	0.132	0.895	0.336	<0.001
Reflective dimension	0.170	1.985	0.049^*^	0.021^*^	0.028
Affective dimension	0.200	2.396	0.018^*^	0.015^*^	0.040
Total score	0.194	2.188	0.021	0.015^*^	0.035
Compassion (SCBCS)	0.205	2.672	0.008^*^	0.015^*^	0.043
Social support (ESS)	0.180	2.338	0.021^*^	0.041^*^	0.033
Social engagement (CSQ)	0.201	2.478	0.014^*^	0.015^*^	0.040

† *Adjusted p-value controlling for adaptive false discovery rate*.

* *p or q < 0.05*.

*3D-WS, Three-Dimensional Wisdom Scale; CSQ, Cognitively Stimulating Questionnaire; ESS, Emotional Support Scale; SCBCS, Santa Clara Brief Compassion Scale; UCLA-3, UCLA Loneliness Scale, Version 3*.

Considering the wide age range of participants and that age was significantly correlated with loneliness (*r* = −0.233, *p* = 0.002) and wisdom total score (*r* = 0.235, *p* = 0.004), we examined whether associations with microbial diversity were moderated by age [i.e., young/middle-aged adults (20–64 years) compared to older adults (>65 years)]. Loneliness significantly interacted with age on alpha-diversity (β = −1.033, *t* = −3.05, *p* = 0.003, *q* = 0.021), such that greater loneliness was associated with lower alpha-diversity in older adults (β = −0.265, *t* = −3.99, *p* < 0.001) but not in young/middle-aged adults (β = −0.006, *t* = −0.013, *p* = 0.91). There was no significant age interaction for wisdom (*p* = 0.118). Similarly, we examined gender as a potential moderating factor, and did not find any significant interactions between gender and psychosocial predictors on alpha-diversity (*p*s > 0.183). Finally, we explored other variables that could potentially explain or moderate the relationship between loneliness and alpha-diversity, including depression and physical well-being. Neither depression (*p* = 0.665) nor physical well-being (*p* = 0.950) was associated with alpha-diversity.

#### Multivariate Analysis

In multivariate analysis, we extracted composite variables from the PLS result and continuously added them into the linear regression model predicting alpha-diversity, examining the contribution of each composite component added by the size of explained variance in the outcome of alpha-diversity (from large to small). Supplementary Table 1 presents results from the linear model predicting alpha-diversity with two PLS components, with age and BMI as covariates. This model was chosen because adding component 3 led to a decreased adjusted *R*^2^. The model revealed that the effect of component 1 was significantly positively associated with alpha-diversity (*p* = 0.008), whereas component 2 was not (*p* = 0.217). Component 1 comprised of a linear combination of all psychosocial predictors (with a negative loading for loneliness and positive loadings for all others, including wisdom, compassion, social support, and social engagement) and accounted for 39.6% of the variance of the psychosocial predictors (Supplementary Figure 1; Supplementary Table 2). The effect sizes of composite predictors were small to medium. In exploratory analyses, neither age nor gender were significant moderators of psychosocial predictors on alpha-diversity (*p*s > 0.238).

### Beta-Diversity

**Figure 2:**
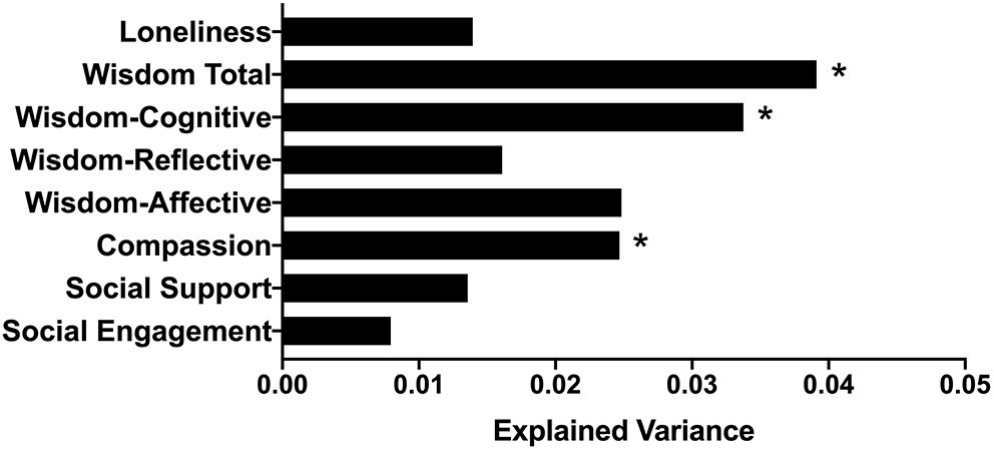
Bar plot indicating the proportion of variance in beta-diversity (Aitchison distance) explained by each psychosocial factor, after controlling for age and BMI. *p or *q* < 0.05.

Beta-diversity was significantly associated with compassion, the cognitive and affective dimensions of wisdom, and wisdom total score (Figure 2). After adjusting for multiple comparisons, compassion, wisdom cognitive dimension, and wisdom total score remained significant. Effect sizes were small to medium. None of the psychosocial predictors interacted with age or gender on beta-diversity (*p*s > 0.055).

## Discussion

To our knowledge, this is the first study to show that loneliness and wisdom, including its important component of compassion, are related to gut microbial diversity and composition. As hypothesized, higher levels of loneliness and lower levels of compassion, wisdom, social support, and social engagement were associated with decreased phylogenetic richness and diversity of the gut microbiome. We further evaluated the multivariate relationship of alpha-diversity with psychosocial variables, and found that the PLS component comprising of all psychosocial variables were significantly associated with alpha-diversity. Wisdom and compassion were associated with both microbial diversity and microbial community structure and composition.

Gut microbial diversity and composition have been previously reported to be associated with personality traits and psychosocial constructs (21–23). Increased gut microbial diversity has been shown to be associated with greater emotional well-being, particularly positive affect, and larger social networks (22, 24). Our results expand upon previous findings, demonstrating that subjective loneliness or perceived social isolation and social support, beyond objective social network size, are associated with and may influence diversity of the gut microbiome. Additionally, we observed a relationship between alpha-diversity and compassion and social engagement, suggesting that pro-social attitudes and activities may positively influence microbial diversity or *vice versa*.

The mechanisms by which loneliness, compassion, and wisdom may be related gut microbial diversity is unknown. It is typically believed that reduced alpha-diversity represents worse physical and mental health, as low microbial diversity has been associated with various diseases, such as obesity, inflammatory bowel disease, and major depressive disorder (35, 36). The relationship between loneliness and microbial diversity is unlikely to be driven solely by physical health or depression, as neither depressive symptoms nor self-reported physical well-being were associated with alpha-diversity in this sample. A species-rich community may be less susceptible to invasion by exogenous pathogens and confer resilience and stability (37). It is possible that loneliness may result in decreased stability of the gut microbiome and, consequently, reduced resistance and resilience to stress-related disruptions, leading to downstream physiological effects such as systemic inflammation. Bacterial communities with low alpha-diversity may not manifest overt disease, but they may be less than optimal for preventing disease. Thus, lonely people may be more susceptible to developing different diseases. In line with our previous work (6, 7), age was negatively correlated with loneliness and positively correlated with wisdom, such that older individuals were less lonely and wiser. However, analyses examining the moderating effect of age revealed that greater loneliness was associated with lower alpha-diversity in older adults, but not young/middle-aged adults, suggesting that older adults may be especially vulnerable to health-related consequences of loneliness, consistent with prior research (67).

On the other hand, social support, compassion, and wisdom may confer protection against loneliness-related instability of the gut microbiome. Prior evidence suggests that perceived social support may buffer the negative effects of chronic stress on pro-inflammatory markers (68). Those effects may be mediated by the gut microbiome. An alternative hypothesis is that the microbiome may help shape social behavior, potentially leading to social isolation and loneliness or contributing to wisdom, which may ward off loneliness. Animal studies suggest that the gut microbiota may influence social behavior and interactions (26–29), although this hypothesis needs to be further explored in humans.

Different dimensions of wisdom were associated with microbiome diversity and composition. Alpha-diversity findings indicate that increased self-reflection, ability to regulate emotions, and pro-social attitudes and behaviors are associated with greater ecological diversity of the gut microbiome within individuals. Beta-diversity findings indicate that compassion and the cognitive dimension of wisdom account for a significant proportion of variance overall microbial community composition differences across individuals.

Our findings have potential clinical implications for developing interventions to reduce loneliness and its health-related consequences. Increasing perceived social support, participation in social activities, and wisdom may improve loneliness by engaging overlapping biological targets. We have shown that it is possible to increase wisdom, particularly its affective and pro-social aspects, with behavioral interventions (69). Prior research also suggests that psychosocial interventions can reduce pro-inflammatory gene expression associated with loneliness (70). Conversely, probiotic and prebiotic interventions targeting the gut microbiome have been shown to reduce cortisol stress response, cognitive reactivity to sadness, and emotional processing bias (71, 72). This evidence presents the exciting possibility that future “psychobiotics” may be a novel therapeutic option for behaviors like loneliness (73).

This study had several important limitations. We did not have a measure of social network size or social interaction. It is important to dissociate subjective loneliness from objective social isolation, because more physical contact and interaction with others would provide increased opportunity to recolonize with more microbes. Additionally, as this was an exploratory study, we did not have measures of medical morbidity, diet, or other biological markers to further investigate relationships with health status. The sample size is modest considering the large age range. The effect sizes in this study were small to medium. A wide range of environmental and genetic factors can affect microbiome composition as well as psychological health. It is possible that some of the genetic and environmental effects on psychological well-being are via influence from the microbiome and *vice versa*. The bi-directionality of gut-brain communications and cross-sectional nature of this study limit interpretations of causality. Loneliness may lead to changes in the gut microbiome or, reciprocally, alterations of the gut milieu may predispose an individual to become lonely. Despite these limitations, the findings represent a step forward in understanding the relationships between the gut microbiome and psychosocial factors that have important consequences for health and well-being. Future longitudinal studies of diverse samples are needed to examine the relationship of changes in loneliness and wisdom with alterations in the gut microbiome as well as other inflammatory, neuroendocrine, and metabolic biomarkers. Similarly, the effects of psychosocial interventions on the microbiota should be examined as well as effects of probiotic treatments on loneliness and wisdom. This type of research will help improve our understanding of the microbiota-gut-brain-axis.

## Data Availability Statement

The datasets presented in this study can be found in online repositories. The name of the repository and accession number can be found below: European Molecular Biology Laboratory's European Bioinformatics Institute (EMBL-EBI) European Nucleotide Archive (ENA), https://www.ebi.ac.uk/ena/browser/home, PRJEB11419. Sample IDs and EBI accession numbers for individual subjects and additional metadata used in this study are available in Supplementary Data. Feature tables can be found in Qiita (https://qiita.ucsd.edu/) under study ID 10317.

## Ethics Statement

The studies involving human participants were reviewed and approved by UCSD Human Research Protections Program. The patients/participants provided their written informed consent to participate in this study.

## Author Contributions

TN: conceptualization, investigation, data curation, formal analysis, visualization, and writing – original draft. XZ, T-CW, JL, and XT: formal analysis, writing – review, and editing. CL: data curation, writing – review, and editing. RK: methodology, software, resources, supervision, writing – review, and editing. DJ: conceptualization, project administration, resources, funding acquisition, supervision, writing – review, and editing. All authors reviewed and approved the final manuscript.

## Conflict of Interest

The authors declare that the research was conducted in the absence of any commercial or financial relationships that could be construed as a potential conflict of interest.
