# Supplementary material for: Association of Loneliness and Wisdom With Gut Microbial Diversity and Composition: An Exploratory Study
Source: Front Psychiatry. 2021 Mar 25;12:648475. doi: 10.3389/fpsyt.2021.648475 (PMC8029068; doi:10.3389/fpsyt.2021.648475)
Supplement: Supplementary file 1 [file Data_Sheet_1.pdf]

Supplementary Material

1 Supplementary Text

1.1 Details of Clinical Assessments

Loneliness was measured using the 20-item UCLA Loneliness Scale (Version 3), which assesses persistent or chronic loneliness with no time reference (1). While the word “lonely” is never used explicitly, respondents rate the frequency of several experiences (e.g., “How often do you feel in tune with others around you?” or “How often do you feel left out?”) on a 4-point scale (options: “I never feel this way”, “I rarely feel this way”, “I sometimes feel this way”, and “I often feel this way”). There are established cut-offs for loneliness severity: total score <28=No/Low, 28 to 43=Moderate, and >43=High (2).

Wisdom was assessed using the 39-item Three-Dimensional Wisdom Scale (3). Wisdom is treated as a latent variable with cognitive, reflective, and affective dimensions. Respondents rate items using a 5-point scale (ranging from “strongly agree” to “strongly disagree”) on statements such as “Ignorance is bliss” (cognitive), “I sometimes find it difficult to see things from another person’s point of view” (reflective), and “Sometimes I feel a real compassion for everyone” (affective).

Compassion was measured using the 5-item Santa Clara Brief Compassion Scale (4), which operationalizes compassion associated with pro-social feelings and behaviors. Respondents record to the extent (“not at all true of me” to “very true of me”) to which they experience different aspects of compassion including general tender feelings towards others, empathy, and compassionate love, which is defined as an altruistic love towards all of humanity (5).

Social support was measured using the Emotional Support Score of the Emotional Support Scale (6). Respondents rate how often their loved ones make them feel “loved and cared for” and are “willing to listen” about worries or problems on a 3-point scale (ranging from “never” to “frequently”).

Depression was measured using the 20-item Center for Epidemiologic Studies Depression Scale (7). Respondents rate the frequency of depressive symptoms capturing depressed mood, feelings of guilt, feelings of worthlessness, psychomotor retardation, loss of appetite, and sleep disturbances on a 4-point scale (ranging from “rarely or none of the time” to “all of the time”).

Physical well-being was measured using the physical component summary score of the 36-item Medical Outcomes Study Health Survey (8-10). The SF-36 assesses eight health concepts: 1) limitations in physical activities because of health problems; 2) limitations in social activities because of physical or emotional problems; 3) limitations in usual role activities because of physical health problems; 4) bodily pain; 5) general mental health (psychological distress and well-being); 6) limitations in usual role activities because of emotional problems; 7) vitality (energy and fatigue); and 8) general health perceptions.

2 Supplementary Tables

Table S1. Coefficients from linear regression model of multivariate partial least squares (PLS) composite variables predicting alpha-diversity (Faith's Phylogenetic Diversity)

	β	***t***	***p***	**Partial η^2**
Component 1	0.717	2.71	0.008*	0.061
Component 2	0.545	1.24	0.217	0.013
Age	-0.015	-0.57	0.569	0.003
BMI	-0.103	-1.32	0.188	0.015

* $p < 0.05$
BMI = body mass index

Table S2. Factor loadings for multivariate PLS composite variables

	**Component 1**	**Component 2**
Loneliness	-0.419	0.272
Wisdom-Cognitive	0.233	-0.836
Wisdom-Reflective	0.462	-0.370
Wisdom-Affective	0.454	-0.140
Compassion	0.417	0.421
Social Support	0.316	0.187
Social Engagement	0.358	0.233

3 Supplementary Figures

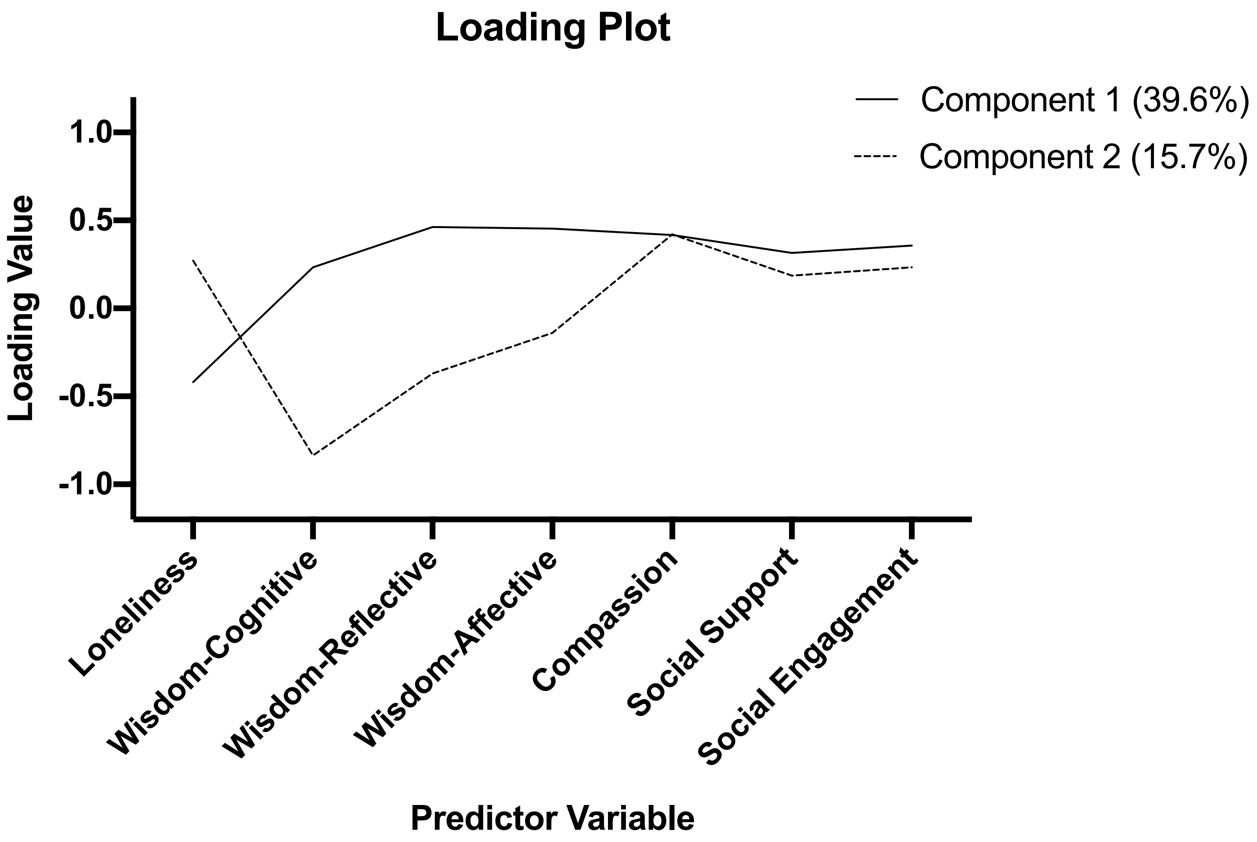

Figure S1. Factor loading plot for multivariate PLS composite variables.

4 References

1. Russell DW. UCLA Loneliness Scale (Version 3): reliability, validity, and factor structure. J Pers Assess. 1996;66(1):20-40.
2. Cacioppo JT, Patrick W. Loneliness: Human nature and the need for social connection. New York: W. W. Norton & Company; 2008.
3. Ardelt M. Empirical Assessment of a Three-Dimensional Wisdom Scale. Research on Aging. 2003;25(3):275-324.
4. Hwang JY, Plante T, Lackey K. The development of the Santa Clara brief compassion scale: An abbreviation of Sprecher and Fehr’s compassionate love scale. Pastoral Psychology. 2008;56(4):421-8.
5. Sprecher S, Fehr B. Compassionate love for close others and humanity. Journal of Social and Personal Relationships. 2005;22(5):629-51.
6. Seeman TE, Lusignolo TM, Albert M, Berkman L. Social relationships, social support, and patterns of cognitive aging in healthy, high-functioning older adults: MacArthur Studies of Successful Aging. Health Psychology. 2001;20(4):243-55.
7. Radloff LS. The CES-D scale: A self-report depression scale for research in the general population. Applied psychological measurement. 1977;1(3):385-401.
8. Ware JE, Sherbourne CD. The MOS 36-item short-form health survey (SF-36): I. Conceptual framework and item selection. Medical care. 1992:473-83.
9. McHorney CA, Ware JE, Raczek AE. The MOS 36-Item Short-Form Health Survey (SF-36): II. Psychometric and Clinical Tests of Validity in Measuring Physical and Mental Health Constructs. Medical Care. 1993;31(3):247-63.
10. Ware J, Kosinski M, Keller S. SF-36 physical and mental health summary scales. A user's manual. 2001:1994.
